# Non-adjacent dependency learning from variable input: investigating the effects of bilingualism, phonological memory, and cognitive control

**DOI:** 10.3389/fpsyg.2023.1127718

**Published:** 2023-07-12

**Authors:** Josje Verhagen, Elise de Bree

**Affiliations:** ^1^Amsterdam Center for Language and Communication, University of Amsterdam, Amsterdam, Netherlands; ^2^Department of Education and Pedagogy, Utrecht University, Utrecht, Netherlands

**Keywords:** statistical learning, variable input, bilingualism, phonological memory, cognitive control

## Abstract

**Introduction:**

One proposed advantage of bilingualism concerns the ability to extract regularities based on frequency information (statistical learning). Specifically, it has been proposed that bilinguals have an advantage in statistical learning that particularly holds in situations of variable input. Empirical evidence on this matter is scarce. An additional question is whether a potential bilingual advantage in statistical learning can be attributed to enhancements in phonological memory and cognitive control. Previous findings on effects of bilingualism on phonological memory and cognitive control are not consistent.

**Method:**

In the present study, we compared statistical learning from consistent and variable input in monolingual and bilingual children (Study 1) and adults (Study 2). We also explored whether phonological memory and cognitive control might account for any potential group differences found.

**Results:**

The findings suggest that there might be some advantage of bilinguals in statistical learning, but that this advantage is not robust: It largely surfaced only in t-tests against chance for the groups separately, did not surface in the same way for children and adults, and was modulated by experiment order. Furthermore, our results provide no evidence that any enhancement in bilinguals' statistical learning was related to improved phonological memory and cognitive control: bilinguals did not outperform monolinguals on these cognitive measures and performance on these measures did not consistently relate to statistical learning outcomes.

**Discussion:**

Taken together, these findings suggest that any potential effects of bilingualism on statistical learning probably do not involve enhanced cognitive abilities associated with bilingualism.

## Introduction

The world's growing bilingual population fuels research into the potential advantages of bilingualism. One proposed advantage concerns statistical learning, or the ability to extract regularities based on frequency information in the input. In this study, we evaluate whether there is a bilingual advantage in statistical learning from linguistic input for children (Study 1) and adults (Study 2). Specifically, we compare statistical learning between monolingual and bilingual speakers from input that is consistent and input that is variable. We also investigate whether any effects of bilingualism on statistical learning are due to enhanced phonological memory and cognitive control.

### Bilingualism and statistical learning

Previous work has found that bilinguals fare better at extracting regularities based on frequency information in the input than monolinguals for various age groups, including 7-month-old infants (Kovács and Mehler, 2009), 14-month-old infants (Antovich and Graf Estes, 2017), 24-month-old toddlers (de Bree et al., 2017), 6- to 12-year-old children and adolescents (Bonifacci et al., 2011), and adults (Benitez et al., 2016; Bulgarelli and Weiss, 2016; Poepsel and Weiss, 2016; Potter et al., 2017; Onnis et al., 2018; for reviews, see Hirosh and Degani, 2017; Bulgarelli et al., 2018; Weiss et al., 2020). Such increased performance has been found for statistical learning from different types of auditory input, including tones, syllables, and Morse words.

However, other studies found no evidence for a bilingual advantage. Yim and Rudoy (2013), for instance, found no difference in performance between monolingual and sequential bilingual 5- to 13-year-olds in visual and auditory statistical learning tasks (see also Bogulski, 2013). Furthermore, in some studies, only partial evidence for a bilingual advantage was found. Bartolotti et al. (2011), for example, compared monolingual and sequential bilingual adults' performance on two learning tasks involving Morse words. In the first, in which Morse words were presented for the first time, bilinguals outperformed monolinguals. In the second, in which Morse code words were presented that conflicted with the words from the first task, performance was unaffected by bilingualism.

Vice versa, de Bree et al. (2017) assessed 24-month-old monolingual and native bilinguals' learning of patterns from auditorily presented nonwords in a condition in which the input was fully consistent and one in which the input was variable, containing 14% of “errors” of the pattern. The patterns in this study involved non-adjacent dependencies: relationships between two elements that are separated by another, intervening element. Non-adjacent dependencies have been studied relatively often in statistical learning and are frequently occurring in natural language in constructions of the type “He is reading.” There were no differences between the monolingual and bilingual toddlers in the consistent input condition, but bilinguals outperformed the monolinguals in the variable condition: the bilingual children showed a stronger sensitivity to the predominant pattern as opposed the other pattern than the monolingual children. Finally, Verhagen and de Bree (2021) found that native bilingual 4- and 5-year-olds fared better than their monolinguals peers on a reaction-time based measure but not an accuracy-based measure in an auditory non-adjacent dependency learning task. There are thus mixed patterns of results regarding effects of bilingualism on statistical learning.

One possible explanation is that bilinguals' enhanced abilities are especially prominent in, or limited to, situations where the input is not uniform, but contains variability (Poepsel and Weiss, 2016). This proposal receives some preliminary support from studies that show that statistical learning from more complex input involving multiple and potentially competing cues is enhanced in bilinguals (Kovács and Mehler, 2009; Bartolotti et al., 2011; Wang and Saffran, 2014; Poepsel and Weiss, 2016; de Bree et al., 2017), but that no such advantage is found in more basic forms of statistical learning. Bilingual learners have to detect the regularities of their two languages on the basis of more limited input than monolingual learners. Furthermore, this input might contain more variation, as the languages might be spoken with more limited linguistic proficiency (Byers-Heinlein and Fennell, 2014). This might mean that bilinguals are better equipped than monolinguals to learn patterns on the basis of more complex and variable cues (Kuo and Anderson, 2012; Kuo et al., 2016). However, to date, no studies have directly compared bilingual children and adults' performance on tasks with variable as opposed to invariable input.

### Cognitive functions

Another open, and potentially related, issue refers to the mechanisms underlying bilinguals' potential advantage (Poepsel and Weiss, 2016; Weiss et al., 2020). Bilinguals' advanced ability could be a direct effect of experience with dual language input, which is typically more complex than single language input as it involves two different language systems (Hirosh and Degani, 2017) with potentially differing quality of input, or an indirect effect, through enhanced cognitive abilities associated with bilingualism (Kovács and Mehler, 2009; Kuo and Kim, 2014; Hirosh and Degani, 2017). These possibilities are not mutually exclusive, as both direct and indirect effects could be at play.

Regarding enhanced cognitive abilities, two functions have been proposed as possible candidates for explaining why bilinguals may learn from variable input more readily than monolinguals: phonological memory and cognitive control (Bartolotti et al., 2011). Phonological memory refers to the ability to store verbal information in short-term memory, and is connected to two processes that are assumed to underlie statistical learning: extraction and integration (Thiessen and Erickson, 2013). Extraction refers to the process of holding statistically congruent clusters in memory (Perruchet and Tillmann, 2010); integration to the process of combining information across these clusters to identify regularities in the input. Verbal working memory, encompassing phonological memory, is considered essential in extraction: participants store exemplars in memory, and integrate information from these prior exemplars (Thiessen, 2017). Features that are consistent across them are strengthened, and features that are inconsistent across them are weakened, leading to knowledge of statistical regularities (Thiessen and Pavlik, 2013). Individual differences in phonological memory have shown positive associations with statistical learning in monolingual children (Kapa and Colombo, 2014) and adults (Karpicke and Pisoni, 2004; Misyak et al., 2010). However, other studies showed no such associations (Kaufman et al., 2010; Siegelman and Frost, 2015; Verhagen and de Bree, 2021).

Cognitive control refers to a set of processes needed to selectively attend to (relevant) stimuli and inhibit or suppress attention to other (less relevant) stimuli. It is typically assessed with tasks in which participants respond to specific targets amongst distracting stimuli or inhibit dominant responses. Cognitive control has been found to predict artificial language learning in monolingual adults and children (Kapa and Colombo, 2014), and might play an even more important role when input is variable: well-developed cognitive control could allow participants to focus their attention selectively on parts of the input, hold partially conflicting information in memory, and suppress less relevant or conflicting information during training and/or at test. In fact, this association between cognitive control and selectively attending to only relevant properties of the language input has been proposed in earlier research (Kuo and Anderson, 2012).

Two studies have tested the suggestion that bilinguals' advantage in statistical learning may be due to enhanced phonological memory and/or cognitive control. Bartolotti et al. (2011) found that cognitive control was positively related to learning Morse code words when word meanings conflicted with meanings learned previously. However, in their study, enhanced cognitive control did not explain bilinguals' advantage on statistical learning; the bilinguals did not outperform the monolinguals on cognitive control. Similarly, in their investigation of auditory non-adjacent dependency learning in monolingual and bilingual kindergarteners, Verhagen and de Bree (2021) found that the bilingual children showed enhanced performance on (part of the) statistical learning measures, but not on a phonological memory task. Furthermore, this study showed that while phonological memory abilities were correlated with statistical learning performance, they did not account for bilinguals' enhanced performance. What remains unknown from these earlier studies, however, is whether phonological memory and cognitive control relate more strongly to statistical learning tasks in which variable input as opposed to consistent input is presented: Bartolotti et al. (2011) did not compare learning from consistent and variable input, even though they had a condition where meanings contrasted with previously learned meanings, and Verhagen and de Bree (2021) looked at statistical learning from consistent input only.

### The present research

It is currently unknown whether bilinguals have an advantage in statistical learning that particularly holds in situations of variable input and, if so, if this advantage can be attributed to enhancements in phonological memory and cognitive control. In the present study, we compare statistical learning from consistent and variable input in monolingual and bilingual children and adults, and explore whether phonological memory and cognitive control might account for any potential group differences found. Note that this second aim is exploratory, as the literature on cognitive advantages of bilingualism is heavily mixed, and the evidence for effects of bilingualism on phonological memory and cognitive control not at all robust (Paap et al., 2015).

## Study 1: children

In Study 1, monolingual and bilingual children were tested on two auditory statistical learning tasks involving non-adjacent dependency patterns: one in which the input was consistent and one in which the input contained exceptions to a predominant pattern, rendering the input variable. We predicted that both groups would be able to learn non-adjacent dependencies from consistent input, based on earlier results showing that very young children are able to do so (Gómez, 2002; Gómez and Maye, 2005). We did not have a prediction for the variable input condition, in the absence of earlier research using similar tasks (except for toddlers, see de Bree et al., 2017). However, we expected that if an advantage for the bilingual participants was found, it would be most prominent for the variable input task (Poepsel and Weiss, 2016). As to relationships with phonological memory and cognitive control, we did not have a clear prediction: while some studies found that these cognitive skills are implicated in statistical learning in both children and adults (Bartolotti et al., 2011; Kapa and Colombo, 2014; de Bree et al., 2017), others did not find such relationships (Verhagen and de Bree, 2021) or failed to show robust effects of bilingualism on these cognitive skills (Paap et al., 2015). Given these mixed findings, we kept this final question exploratory.

## Method

### Participants

Participants were 53 children with a mean age of 8;5 years (*SD* = 1;1, min-max = 6;9−10;9). We based our sample size on earlier studies with similar designs and age ranges that found effects of bilingualism on statistical learning. These had similarly sized samples as ours (Bartolotti et al., 2011; Wang and Saffran, 2014) or smaller samples (Poepsel and Weiss, 2016; de Bree et al., 2017). Children between 6 and 10 years were recruited, because earlier work had shown that children of this age range can conduct the non-adjacent dependency task used in our study (Hakvoort, 2009) and because children in this age range are in the same, primary school, phase as the participants in Bonifacci et al. (2011) and Yim and Rudoy (2013). 25 children were monolingual (mean age: 8;4 years, *SD* = 1;0) and 28 were bilingual (mean age: 8;5 years, *SD* = 1;2). Age did not differ between the groups [*t*_(1, 51)_ = 0.061, *p* = 0.952, *d* = 0.017]. The monolingual group contained 13/26 (50%) boys and the bilingual group contained 11/28 (39%) boys. This difference in gender was not significant (χ^2^(54) = 0.627, *p* = 0.429).

Children had been recruited through primary schools offering either bilingual or monolingual education in the Netherlands as well as through personal contacts, and the participant database of the [Utrecht University Babylab]. The monolingual children all came from monolingual Dutch homes and had not been in regular contact with another language than Dutch, as indicated in a parent questionnaire. The bilingual children learned Dutch as well as one out of a diverse set of other languages at home: English (*n* = 9), Turkish (*n* = 4), Russian (*n* = 3), Armenian (*n* = 3), German (*n* = 2), Spanish, Italian, Sranan Tongo, French, Limburgian, Bulgarian, Romanian (all *n* = 1). All children had been exposed to their other language from birth. Exposure to each language varied, as indicated by parents' responses in the questionnaire that were available for 20 out of 28 children: 12 children were mostly exposed to their other language and sometimes to Dutch; 6 children were mostly exposed to Dutch and sometimes to their other language, and 2 children were equally exposed to each language. Two children were multilingual as they spoke two languages other than Dutch at home (one child spoke Dutch, English, and Romanian; another child spoke Dutch, Italian and Swedish). Dutch receptive vocabulary, as indicated through children's raw scores on the Dutch Peabody Picture Vocabulary Test (PPVT-III-NL, Dunn and Dunn, 2005), [*M* = 113.44, *SD* = 11.26 for monolinguals; *M* = 109.82, *SD* = 16.04 for bilinguals] and controlled for age, did not differ significantly between the groups, *F*_(1, 50)_ = 1.281, *p* = 0.263). One monolingual child performed only the consistent input NADL experiment; two children (one monolingual, one bilingual) performed only the variable input NADL experiment, due to illness. Written informed consent was obtained from children's parents.

### Materials

#### Non-adjacent dependency learning (NADL) experiments

##### Consistent input NADL

In the consistent input NADL experiment, participants listened to a miniature artificial language. This was modeled after the languages used in previous studies on non-adjacent dependency learning in English children and adults (Gómez, 2002; Gómez and Maye, 2005) and the same as in de Bree et al. (2017). Prior to the experiment, children were told that they were going to listen to a robot that would speak an odd language and informed that they should pay attention to the ordering of the elements in the language. This instruction was included based on previous studies with similar aged groups (Hakvoort, 2009) showing that children otherwise did not understand the task. Children colored a robot while listening to the artificial language (Saffran et al., 1997; Grama et al., 2016).

The language was presented on a laptop computer and through headphones. It consisted of three-item strings that took the form a-X-b or c-X-d. The elements a, b, c, and d represented the novel words *rak, toef*, *sot* and *lut*, and X was drawn from a pool of 24 novel words (*wadim, kasi, poemer, kengel, domo, loga, gopem, naspu, hiftam, dieta, vami, snigger, rogges, densim, fidang, rajee, seeta, noeba, plizet, banip, movig, sulep, nilbo*, and *wiffel)*. A set size of 24 X-elements was chosen because this yielded the strongest learning effects in previous studies (Gómez and Maye, 2005; Hsu et al., 2014). In the training phase of the experiment, participants were either presented to language 1 that contained the triplets a-X-b and c-X-d (i.e., *rak*-X-*toef*, *sot*-X-*lut*) or to language 2 that contained the triplets a-X-d and c-X-b (i.e., *rak*-X-*lut, sot*-X-*toef*). These two language versions were used to rule out any potential effects of the phonological properties of the stimuli or stimuli combinations. There were seven iterations of each of the 48 triplets (2 dependencies per language ^*^ 24 X-elements), resulting in a total of 336 triplets per language (see Table 1). The training phase lasted about 15 mins.

**Table 1:** Stimuli of the training phase in the consistent and variable input NADL experiments.

**Experiment**	**Number of triplets**	**Language1**		**Language2**	
Consistent input	336	a-X_(1 − 24)_-b	c-X_(1 − 24)_-d	a-X_(1 − 24)_-d	c-X_(1 − 24)_-b
		(rak X toef)	(sot X lut)	(rak X lut)	(sot X toef)
Variable input	288	a-X_(1 − 24)_-b	c-X_(1 − 24)_-d	a-X_(1 − 24)_-d	c-X_(1 − 24)_-b
		(rak X toef)	(sot X lut)	(rak X lut)	(sot X toef)
	48	a-X_(1 − 24)_-d^*^		a-X_(1 − 24)_-b^*^	
		(rak X lut)^*^		(rak X toef)^*^	

^*^Refers to “noise” triplets. X refers to the different X-items used.

Triplets had been created on the basis of novel words that were spliced from triplets recorded from a female native speaker of Dutch, and subsequently, combined into new triplets for both languages. Consequently, the two languages did not differ in pronunciation and there were no speaker-induced differences in individual triplets. A 250-ms inter-stimulus interval occurred in between the three nonwords of each triplet. To ensure that the three nonwords were perceived as one triplet, a 750-ms interval occurred between triplets. In the test phase of the experiment that directly followed the training phase, a forced-choice selection task was presented, in which participants listened to eight pairs of triplets. Each pair contained two spoken sentences that were played in turn: one from the language presented during training (trained triplet) and one from the other language (untrained triplet) (see Appendix Table A1). Participants were asked to indicate for each pair which triplet matched the language they had listened to in the training phase by pressing one of two response buttons on a laptop keyboard. Only the X-elements *wadim, kasi, poemer* and *rogges* were used in the test phase. Pairs were presented in pseudo-randomized lists in which no more than two elements of the same type were presented consecutively and the ordering of triplets within pairs was counterbalanced across participants. Throughout the experiment, stimuli presentation and response logging were controlled through E-prime 2.0 (Psychology Software Tools).

##### Variable input NADL

This experiment was the same as the consistent input NADL experiment, except that a portion of the triplets was inconsistent with the logic of the artificial language. Specifically, 48 out of 336 triplets (14%) contained “errors” in that they came from the other language. For example, if participants were trained on language 1 (i.e., *rak*-X-*toef*, *sot*-X-*lut*), they would hear 48 instances of incorrect *rak*-X-*lut* from language 2 that were intermixed with the correct triplets from language 1. These “errors” were randomly picked from a list and inserted at fixed, pseudo-random positions in the training. Following de Bree et al. (2017), for only one of the two dependencies, an alternative was presented in which the structure had been disrupted. So, in language 1, participants were presented with *rak-X-toef* and *sot-X-lut* as the predominant pattern (86% of the items) and with incorrect ^*^rak-X-lut in 14% of the items (see Table 1).

As in the consistent input experiment, the forced-choice selection task in the test phase of the experiment contained eight item pairs. Four of these involved a contrast between a triplet from the training phase (trained triplet) and a triplet from the other language (untrained triplet) and thus were identical to the test pairs in the consistent input NADL experiment. The other four involved a contrast between a trained triplet and a “noise triplet,” and thus involved a contrast between items that had both been presented during the test phase, but with different frequencies. The first type of item was included to assess learning of the non-adjacent dependency rules, but under more challenging conditions than in the consistent input experiment. The second type was included to see whether participants could identify the more frequent triplet. These items were not included to address directly our research question on whether participants would learn the predominant pattern, but to see if participants could distinguish between the two triplets. This would signal that they were sensitive to the relative frequencies of both types of triplet. As in the consistent input experiment, only four X-elements were used during the test phase, and items were presented in pseudorandomized and counterbalanced lists. Specifically, no more than two trained-untrained items or trained-noise items were presented after one another and the order of triplets within items was counterbalanced across participants.

#### Nonword repetition

The nonword repetition (NWR) task by Rispens and Baker (2012) was used to assess phonological memory. In this task, children hear a prerecorded nonword over headphones and are then asked to repeat it. The task contains 40 items that range between two and five syllables (ten of each type). Items are pseudo-randomly divided into two blocks of twenty items, with a short break in between. Children's responses were recorded and coded as (in)correct. Cronbach's alpha was.77. Scores were computed as the number of correct responses (maximum score: 40).

#### Flanker task

Cognitive control was assessed with a Dutch version of the Flanker task used by Engel de Abreu et al. (2012), in which horizontal rows of five equally spaced yellow fish are presented on a laptop screen. Children have to indicate the direction of the central fish by pressing the corresponding left or right response button on each side of the laptop keyboard as quickly as possible. On congruent items (50% of items), the flanking fishes point in the same direction as the central fish. On incongruent items (50% of items), the flanking fishes point in the opposite direction. Each item starts with a 1-second fixation cross in the middle of the screen, followed by the fish array for five seconds or until a response is made. Responses are followed by a 400-ms blank interval. There are two blocks of 20 items each with randomized presentation of congruent and incongruent items, preceded by eight practice items. Reaction times and accuracy were recorded through E-Prime 2.0 (Psychology Software Tools).

### Procedure

Children were tested individually twice by a research assistant in a quiet room at home or school. The sessions were about 40 mins each, with at least one to two weeks in between. The order of the NADL experiments was counterbalanced across sessions. Twenty-five children performed the consistent input experiment in the first session (11 bilinguals; 14 monolinguals); 28 children performed the variable input experiment in the first session (17 bilinguals; 11 monolinguals). Tasks were presented in a fixed order within sessions: the consistent input experiment preceded the Flanker task, and the variable input experiment preceded the NWR task. Children received a sticker after each task and a small gift at the end of the session.

Written informed consent was obtained from the parents before testing; consent and participation could be retracted at any time. The research was conducted in accordance with American Psychological Association ethical standards as well as The Netherlands Code of Conduct for Scientific Practice issued in 2004 (revised in 2018 by the Association of Universities in The Netherlands).

### Analyses

We first checked whether performance on the NADL experiments was significantly above chance in each group through *t*-tests against the 50% chance level. Then, we ran a generalized linear-mixed effect regression model on participants binary scores (correct vs. incorrect) in the forced-choice selection tasks in each experiment, using the *lme4* package (Bates et al., 2015) in R version 3.4.1 (R Core Team, 2017). As fixed effects, we included “group” (monolingual vs. bilingual), “version” (consistent vs. variable), “experiment order” (consistent input experiment first vs. second), and “age” (in years). Effects of “language” (language 1 vs. language 2) were explored, but not retained, because this factor did not have an effect and yielded a less well-fitting model, as indicated by a higher AIC-value. By-item random intercepts were included, to obtain the maximal random effect structure supported by the data. As a further exploratory analysis, we ran a similar model on the data of the variable input experiment only, to see whether group interacted with item type (trained-untrained pairs vs. trained-noise pairs). This analysis was included to yield a more complete picture of participants' knowledge of the relative frequencies of the dependencies presented in the variable input experiment.

To assess whether individual differences in phonological memory and cognitive control related to participants' learning of the dependency relations in the two groups, we first excluded reaction times below 200 ms and above three standard deviations of children's individual means (<1.8% of all items) for the Flanker task, following Engel de Abreu et al. (2012). Also, following Engel de Abreu et al., accuracy scores were computed, but not analyzed because they were at ceiling (95% correct or higher). Mean reaction times on correct items were calculated for the (in)congruent items separately. Next, we tested for effects of group on participants' scores on the NWR and Flanker task through a *t*-test and a linear model with item type (congruent vs. incongruent) and group as fixed effect factors and by-subject random intercepts, respectively. Subsequently, we calculated bivariate correlations between scores on the NWR and Flanker task and summed accuracy scores in the NADL experiments for the monolinguals and bilinguals separately. Finally, to examine how phonological memory and cognitive control related to statistical learning as well as any effects of bilingualism on statistical learning, we ran the same model as above, with the NWR and Flanker scores as additional fixed effect scores. In all mixed-effect models, orthogonal sum-to-zero contrast coding was applied to our fixed effects “group” (bilinguals: −1/2, monolinguals +1/2), “experiment version” (consistent: −1/2, variable: +1/2) and “experiment order” (consistent input first: −1/2, consistent input last: +1/2) (Schad et al., 2020). Continuous predictors were centered around zero. All data files and scripts can be found at: https://osf.io/b4ps6/?view_only=a18f5b5cb1d04905b6c26f29de2f43b1.

## Results

### Results for NADL experiments

Descriptive statistics for the two NADL experiments are presented in Table 2.

**Table 2:** Descriptive statistics for the forced-choice selection tasks in the NADL experiments.

		**Monolinguals**		**Bilinguals**	
**Experiment**	**Item type**	**M**	**(SD)**	**M**	**(SD)**
Consistent input	Trained—untrained	0.53	(0.22)	0.63	(0.17)
Variable input	Trained—untrained	0.46	(0.23)	0.47	(0.24)
	Trained—noise	0.45	(0.25)	0.43	(0.24)

*T*-tests comparing against the 50%-chance level showed that, in the consistent input experiment, the bilingual children performed above chance, but the monolingual children did not [monolinguals: *t*_(1, 23)_ = 0.636, *p* = 0.531, *d* = 0.130; bilinguals: *t*_(1, 25)_ = 3.844, *p* = 0.001, *d* = 0.754]. In the variable input experiment, neither of the groups performed above chance, neither on the trained-untrained trials [monolinguals: *t*_(1, 22)_ = −0.890, *p* = 0.383, *d* = −0.189; bilinguals: *t*_(1, 26)_ = −0.593, *p* = 0.558, *d* = −0.114] nor on the trained-noise trials [monolinguals: *t*_(1, 23)_ = −1.045, *p* = 0.307, *d* = −0.213, bilinguals: *t*_(1, 26)_ = −1.615, *p* = 0.118, *d* = −0.331].

A generalized linear mixed-effect model with “group,” “experiment version,” and “experiment order” as fixed effects, and “age” as a fixed effect control factor, showed no main effect of group (β = −0.250, SE = 0.179, *z* = −1.400, *p* = 0.162) or experiment order (β = −0.066, SE = 0.179, *z* = −0.367, *p* = 0.714). A main effect of experiment version indicated that children performed better on the consistent than variable input experiment (β = −0.412, SE = 0.184, *z* = −2.245, *p* = 0.025). There also was an interaction effect between group, experiment version and experiment order (β = 1.624, SE = 0.717, *z* = 2.267, *p* = 0.023), which indicated that the difference in performance across the two experiment versions was larger for the bilinguals than monolinguals and interacted with experiment order: for the bilinguals, the difference was largest when they performed the variable input experiment first, while for the monolinguals it was largest when they performed the consistent input experiment first. For descriptives per experiment plotted by experiment order (see Appendix Figure B1). The other effects and interactions were not significant (see Appendix Table B1). A model on children's scores on the scores on the variable input experiment only with “item type” (trained-untrained vs. trained-noise) showed no effects (see Appendix Table B2).

### Statistical learning and relationships with phonological memory and cognitive control

Descriptive statistics for the NWR and Flanker tasks are presented in Table 3. Data were available for all children, except one monolingual child.

**Table 3:** Descriptive statistics for the nonword repetition task and flanker task.

	**Monolinguals**		**Bilinguals**	
	***M***	***(SD)***	***M***	***(SD)***
***Nonword repetition task***				
Number correct (maximum = 40)	26.32	(5.48)	28.71	(5.93)
***Flanker task***				
RT congruent items	860.12	(301.76)	1008.81	(353.41)
RT incongruent items	952.66	(355.67)	1113.16	(435.31)
RT difference score	92.54	(116.87)	104.34	(131.86)

The numerically slightly higher NWR scores of the bilingual children than the monolingual children were not significantly different [*t*_(1, 50)_ = 1.529, *p* = 0.133, *d* = 0.419]. Regarding the Flanker scores, a linear model with item type (congruent vs. incongruent) and group as fixed effect factors and by-subject random intercepts showed an effect of item type (β = 98.44, SE = 17.41, *t* = 5.654, *p* < 0.001), indicating that children responded more slowly to the incongruent than congruent items, but no effect of group (β = −154.60, SE = 100.69, *t* = −1.535, *p* = 0.131) or interaction between item type and group (β = −11.80, SE = 34.83, *t* = −0.339, *p* = 0.736).

In the absence of effects of group, it was unlikely that differences in phonological memory and cognitive control could account for the slight advantage of the bilingual children in NADL—which was only observed in the *t*-tests across chance level in the consistent input experiment. Yet, to rule out this possibility, we calculated partial (age-controlled) correlations between the scores in the NADL experiments and the NWR and Flanker scores. Correlations between NWR and NADL were weak and non-significant. Monolinguals' performance on the Flanker task (incongruent trials and difference score) correlated negatively with performance in the variable input experiment, indicating that children who performed relatively well on the Flanker task had relatively good performance in this experiment. For the full correlation matrix (see Appendix Table B3).

On the basis of these data, it seems unlikely that phonological memory and cognitive control played a major role in the bilinguals' higher performance in the variable input experiment. Indeed, adding the NWR and Flanker scores as additional fixed-effect factors to our previous mixed-effect model yielded no effects of NWR (β = 0.006, SE = 0.009, *z* = 0.655, *p* = 0.512) or Flanker scores (β = 0.071, SE = 0.075, *z* = 0.956, *p* = 0.339). The previously found effect of version remained, indicating that children obtained higher scores in the consistent input than variable input experiment, irrespective of group (β = −0.410, SE = 0.185, *z* = −2.219, *p* = 0.027), as did the interaction between group, experiment version and experiment order (β = 1.635, SE = 0.722, *z* = 2.265, *p* = 0.024). For the full results (see Appendix Table B4).

## Summary study 1

We investigated whether bilingual children showed enhanced statistical learning, particularly in learning from variable input. Our results (see an overview in **Table 6**) suggested better performance for the bilinguals only in the consistent input experiment, but only through *t*-tests. In a mixed-effect regression analysis, there were no effects of group and no interaction between group and experiment. Instead, a complex interaction between group, experiment version and experiment order was found that we will turn to in the Discussion.

We also assessed whether a potential statistical learning advantage in learning from variable input was due to potentially better performance on phonological memory (NWR) and cognitive control (Flanker) in the bilingual group. However, the bilingual children did not show better performance on the NWR and the Flanker task than the monolingual group. Furthermore, there was no association between statistical learning and the cognitive abilities (NWR and Flanker).

## Study 2: adults

In Study 2, we investigated the same questions as in Study 1, in adults. We predicted that both monolingual and bilingual adults would be able to learn non-adjacent dependencies from consistent input, as evidenced by above-chance performance, based on earlier results for English-speaking adults (Gómez, 2002; Gómez and Maye, 2005) and Dutch-speaking adults (Grama et al., 2016). Furthermore, we initially predicted that any advantage for the bilinguals would be most prominent for the variable input task (Poepsel and Weiss, 2016). Given that this prediction was not borne out for the children in Study 1, we were not sure what to expect for the adults. Regarding phonological memory and cognitive control, we had no clear predictions either, given that our already tentative prediction in Study 1 was not supported by the data.

## Method

### Participants

Participants were 54 adults with a mean age of 26;0 years (*SD* = 0;6, min-max = 19–37). Of these, 26 were monolingual Dutch and 28 were bilingual (Dutch + other language). As in Study 1, sample size was based on earlier studies with similar test designs that attested effects of bilingualism on statistical learning and had similarly sized samples (Bartolotti et al., 2011; Wang and Saffran, 2014) or smaller samples (Poepsel and Weiss, 2016; de Bree et al., 2017). Participants were recruited via research assistants' friends, acquaintances, and families. They were classified as monolingual if they used only Dutch at home and did not speak another language than Dutch with friends or families regularly. Participants were classified as bilingual if they spoke Dutch and another language(s) at home on a daily basis, with friends/families or at work. The bilingual participants spoke one out of a set of the following languages, next to Dutch: Armenian (*n* = 16), English (*n* = 4), German (*n* = 4), Arabic (*n* = 1), Spanish (*n* = 1), French (*n* = 1), Hebrew (*n* = 1). Participants reported high proficiency levels in Dutch, as rated on a five-point scale ranging from 1 “zero proficiency” to 5 “fluent,” with an average of 4.33 (*SD* = 0.80) for questions assessing speaking and listening.

For the bilinguals, self-reported proficiency in the other language was also generally high (*M* = 4.80, *SD* = 0.57). Three participants reported higher proficiency in Dutch than their other language; the remaining participants reported equally high proficiency or higher proficiency in their other language. Twenty of the bilingual participants had acquired their other language prior to Dutch, six had acquired Dutch first, and two bilinguals had acquired both languages simultaneously from birth. For the 26 bilinguals who had learned their languages successively, sixteen had acquired their second language before the age of twelve. Four participants used more than two languages on a daily basis at home (*n* = 2 Dutch/Armenian/Russian, *n* = 2 Dutch/Armenian/Arabic). Although these latter participants were thus multilingual rather than bilingual, we refer to them as bilinguals in this study.

Mean ages [monolingual: 25;10 years (*SD* = 0;5); bilingual: 26;3 years (*SD* = 0;7)] did not differ significantly between the groups [*t*_(52)_ = 0.262, *p* = 0.794, *d* = 0.072]. The distribution of sex (monolingual: 8/26 (31%) males; bilingual:14/28 (50%) males) did not differ either (χ^2^(54) = 2.065, *p* = 0.151). Participants' mean highest level of education, as established on a scale with “1” (primary school) to “6” (university) as its scale points, did not differ significantly between groups (monolingual: *M* = 5.00 (*SD* = 1.35), available for 25/26 monolinguals; bilingual: *M* = 4.68 (*SD* = 1.36), available for 28/28 participants)—[*t*_(1, 51)_ = 0.860, *p* = 0.384, *d* = 0.237]. One monolingual participant and two bilingual participants performed only the variable input experiment, due to illness.

### Materials

#### NADL experiments

##### Consistent input NADL

This experiment was the same as the consistent input experiment in Study 1, except for the instructions; participants were told that they were going to listen to an odd language, and informed that they had to answer some questions about the language later on. During listening, participants colored a mandala.

##### Variable input NADL

This experiment was the same as the variable input experiment used in Study 1, except for the instructions, which were the same as in the consistent input experiment for the adults.

#### Phonological memory and cognitive control

##### Nonword repetition

The Dutch Nonword Repetition Test (NRT) was administered to assess participants' phonological memory ability (De Jong, 1998). In this test, participants repeat pre-recorded nonwords. The test contains two practice items and 48 nonwords that vary in length from two to five syllables (twelve nonwords of each type). The audio files were implemented in the experimental software E-prime 2.0 and administered to participants on a laptop via headphones. Responses were coded as (in)correct, and scores were computed as the number of correct responses (maximum = 48). Cronbach's alpha was 0.88.

##### Trail making test

The Trail Making Test (TMT) was used to assess cognitive control (Reitan, 1956). In part A of this test, subjects are asked to draw lines to connect 25 circles containing numbers (1–25) distributed over a sheet of paper in ascending order. In part B, the circles contain both numbers (1–13) and letters (A-L), and subjects are asked to connect the circles in ascending order, while alternating between numbers and letters (1-A-2-B-3-C, etc.). Participants are instructed to do this as fast as possible, without lifting their pen from the paper. Scores are: (i) the time in seconds it takes participant to connect the “trail” in part A, (ii) the time in seconds that it takes to connect the “trail” in part B, and (ii) the difference between the scores for parts B and A. Part A mainly assesses visuo-perceptual processing, part B primarily working memory and secondarily task switching, and the B-A difference score cognitive control (Sánchez-Cubillo et al., 2009).

### Procedure

Participants were assessed individually at a quiet place at their home or university in two sessions that were one and two weeks apart. Administration of the consistent and variable input experiments was counterbalanced across sessions. Thirty participants performed the consistent input experiment first (15 monolinguals; 15 bilinguals); 24 participants performed the variable input experiment first (13 bilinguals; 11 monolinguals). Task order was fixed within sessions: the consistent input experiment preceded the TMT, and the variable input experiment preceded the NRT.

Written informed consent was obtained from the participants before testing; consent and participation could be retracted at any time. The research was conducted in accordance with American Psychological Association ethical standards as well as The Netherlands Code of Conduct for Scientific Practice issued in 2004 (revised in 2018 by the Association of Universities in The Netherlands).

### Analyses

To assess the two groups' performance on the NADL experiments, the same analyses were performed as in Study 1: We first conducted *t*-tests against the 50% chance level on the two NADL tasks separately. We then ran a generalized linear-mixed effect regression model on participants accuracy scores (correct vs. incorrect) in the forced-choice selection tasks with “group” (monolingual vs. bilingual), “experiment” (consistent input vs. variable input), and “experiment order” (consistent input first vs. consistent input last) as fixed effects. By-subject and by-item random intercepts were included. We also ran a similar model on the data of the variable input experiment only, to test for effects of group on participants' performance on the trained-untrained as opposed to the trained-noise items.

To address our second question of how individual differences in phonological memory and cognitive control related to participants' learning scores, we performed the same analysis as in Study 1 (i.e., testing for group effects on NWR and TMT; bivariate correlations between NWR/TMT, and NADL scores; mixed-effect regression with NWR and TMT scores as fixed effect factors). All data files and scripts can be found at: https://osf.io/b4ps6/?view_only=a18f5b5cb1d04905b6c26f29de2f43b1.

## Results

### Results for NADL experiments

Descriptive statistics for both NADL experiments are presented in Table 4.

**Table 4:** Descriptive statistics (proportions correct) for the NADL experiments.

		**Monolinguals**		**Bilinguals**	
**Experiment**	**Item type**	**M**	**(SD)**	**M**	**(SD)**
Consistent input	Trained-untrained	0.64	(0.26)	0.63	(0.23)
Variable input	Trained-untrained	0.56	(0.28)	0.63	(0.28)
	Trained-noise	0.51	(0.26)	0.53	(0.28)

*T*-tests comparing performance against the 50% chance level showed that, in the consistent input experiment, both groups performed above chance [monolinguals: *t*_(1, 23)_ = 2.781, *p* = 0.012, *d* = 0.555; bilinguals: *t*_(1, 24)_ = 2.783, *p* = 0.010, *d* = 0.557]. In the variable input experiment, the monolinguals did not perform above chance when presented with trained and untrained triplets [*t*_(1, 25)_ = 1.100, *p* = 0.282, *d* = 0.216], but the bilinguals did [*t*_(1, 25_) = 2.409, *p* = 0.024, *d* = 0.472]. On the items in the variable input experiment that involved a contrast between a trained and a noise triplet, neither of the two groups performed above chance [monolinguals: *t*_(1, 25)_ = 0.189, *p* = 0.852, *d* = 0.037, bilinguals: *t*_(1, 25)_ = −0.531, *p* = 0.600, *d* = 0.107]. A generalized linear mixed-effect model on the correct/incorrect scores on the “trained-untrained” items with “group,” “experiment version” and “experiment order” as fixed effects showed no main effects of group (β = −0.020, SE = 0.310, *z* = −0.065, *p* = 0.948), experiment version (β = −0.311, SE = 0.212, *z* = −1.466, *p* = 0.143) or experiment order (β = −0.272, SE = 0.309, *z* = −0.879, *p* = 0.379). The interaction between group and experiment version was not significant either (β = −0.745, SE = 0.401, *z* = −1.858, *p* = 0.063). The only effect found was a three-way interaction between group, experiment version and experiment order (β = −1.880, SE = 0.803, *z* = −2.341, *p* = 0.019). Monolinguals showed a larger difference in performance between the consistent and variable input experiment than the bilinguals and this interaction was related to experiment order: for the monolinguals, the difference in performance across versions was largest when they performed the variable input experiment first, whereas order did not matter for the bilinguals (see Appendix Figure C1). For the full results of the model (see Appendix Table C1). An analysis on the variable input experiment only in which “item type” was included and “experiment version” and “experiment order” were left out showed no main effects of group (β = −0.226, SE = 0.231, *z* = −0.977, *p* = 0.328), item type (β = −0.347, SE = 0.215, *z* = −1.613, *p* = 0.107), and no interaction between group and item type (β = 0.233, SE = 0.407, *z* = 0.571, *p* = 0.568) (see Appendix Table C2).

### Statistical learning and relationships with phonological memory and cognitive control

Descriptive statistics for the NRT and TMT are presented in Table 5. NRT scores were missing for four participants due to illness (*n* = 2) or experiment error (*n* = 2); TMT scores were missing for one (Hebrew-Russian-Dutch-speaking) participant who had not automatized the alphabet and therefore had trouble completing part B of the task in which numbers and letters had to be connected in alternating order (1-A-2-B-3-C-4-D etc.).

**Table 5:** Descriptive statistics for the nonword repetition test and trail making test.

	**Monolinguals**		**Bilinguals**	
	**M**	**(SD)**	**M**	**(SD)**
**Nonword repetition test (NRT)**				
Number correct (max = 48)	33.84	(5.74)	34.08	(7.75)
**Trail making test (TMT)**				
Part A (time in sec.)	18.96	(7.10)	26.89	(8.81)
Part B (time in sec.)	41.23	(19.54)	53.70	(19.58)
B—A difference score	22.27	(15.61)	26.81	(14.37)

The NRT scores did not differ between groups [*t*_(1, 49)_ = 0.125, *p* = 0.902, *d* = 0.035]. On the TMT, the monolinguals were significantly *faster* than the bilinguals on both parts of the test [part A: *t*_(1, 51)_ = 3.614, *p* < 0.001, *d* = 0.991, part B: *t*_(1, 51)_ = 2.231, *p* = 0.024, *d* = 0.637]. They also obtained slightly lower TMT difference scores than the bilinguals, but this difference was not significant [*t*_(1, 51)_ = 1.102, *p* = 0.276, *d* = 0.303].

These outcomes rendered it unlikely that bilinguals' slightly enhanced performance on the variable input experiment, visible only through above-chance performance, could be attributed to differences in phonological memory and cognitive control. However, to see how these cognitive skills related to statistical learning performance, we explored the bivariate correlations between the NRT and TMT scores and the scores on the NADL experiments, and added the NRT and TMT scores to the regression model above. The correlation matrix showed a significant moderate correlation between the TMT difference scores and performance on NADL consistent; participants with a smaller TMT difference score (indicating better cognitive control) tended to perform better on NADL consistent input. For the variable input experiment, no significant correlations were found in either group. For the full correlation matrix (see Appendix Table C3). When the NRT and TMT scores were added to the mixed-effect model presented above, as additional fixed-effect factors, there were still no effects of “group” (β = −0.301, SE = 0.303, *z* = −0.995, *p* = 0.320) or experiment version (β = −0.363, SE = 0.219, *z* = −1.657, *p* = 0.098). However, there was an effect of experiment order (β = −0.673, SE = 0.307, *z* = −2.193, *p* = 0.028), indicating that accuracy was higher when the inconsistent input experiment was presented first. The interaction effect between “group” and “experiment version” was now also significant (β = −0.892, SE = 0.416, *z* = −2.156, *p* = 0.031), indicating that the effect of group was largest for the variable input experiment. The above-found three-way interaction between “group,” “experiment version” and “experiment order” remained (β = −1.733, SE = 0.831, *z* = −2.085, *p* = 0.037). There was no effect of NRT on NADL performance (β = −0.016, SE = 0.015, *z* = −1.070, *p* = 0.285). There was a negative effect of the TMT difference score on NADL performance (β = −0.237, SE = 0.106, *z* = −2.232, *p* = 0.026), indicating that participants with well-developed cognitive control were generally better in distinguishing between trained and untrained items in the NADL experiments. For the full model results (see Appendix Table C4).

## Summary study 2

The main findings of Study 2 are summarized in Table 6. The first aim of this study was to assess whether bilingual adults show an advantage in statistical learning particularly in learning from variable input. The results we found were mixed: while only the bilingual group showed above-chance performance in the variable input experiment, the interaction between group and experiment version did not surpass the 0.05 alpha level in a regression analysis, unless scores on phonological memory (NRT) and cognitive control (TMT) were added to the analysis. Furthermore, an interaction between group, experiment version and experiment order was obtained, that we will return to in the Discussion.

**Table 6:** Overview of statistically significant effects in study 1 and study 2.

		**Study 1: children**	**Study 2: adults**
**Task**	**Items**	**Results**	**Results**
NADL consistent	Trained—untrained	Bilinguals performed above chance.	Monolinguals and bilinguals performed above chance.
NADL variable	Trained—untrained	–	Bilinguals performed above chance.
	Trained—noise	–	–
		Main effect of version: Participants generally scored higher on consistent than variable input experiment. Group ^*^ Version ^*^ Order Larger difference in performance between the experiment versions for bilinguals than monolinguals: for bilinguals, the difference was largest when variable input was first; for monolinguals when consistent input was first.	Group ^*^ Version ^*^ Order: Larger difference in performance between the experiment versions for monolinguals than bilinguals: for monolinguals, the difference was largest when variable input was first; for bilinguals, there was no difference in performance depending on experiment order.
Phonological memory (NWR)		–	–
Cognitive control (TMT/Flanker task)		–	Monolinguals outperformed bilinguals.
		–	Higher cognitive control was positively associated with better statistical learning.

The second aim of this study was to evaluate whether a potential statistical learning advantage in learning from variable input was due to enhanced phonological memory and cognitive control in the bilingual group. However, the bilingual group did not outperform the monolingual group on either NRT or TMT and there was only a positive association between TMT and NADL performance in the consistent input experiment. Adding the NRT and TMT scores to the regression model did not change the above results, except that the trend for an interaction between group and version in the model without NRT and TMT scores became significant. It also showed that participants with better cognitive control were more likely to perform better on the NADL experiments, regardless of group and type of input.

## General result summary

The main findings of Study 1 and Study 2 are summarized in Table 6.

## Discussion

We conducted two studies, one with children and one with adults, to target two questions. The first was whether bilinguals would display enhanced statistical learning, specifically in learning from variable input. The second was whether better statistical learning (from variable input) would be related to improved phonological memory and cognitive control. In both studies, statistical learning was assessed through a non-adjacent dependency learning task.

### Statistical learning in monolinguals and bilinguals

Our results for the children (Study 1) showed that only the bilingual children performed significantly above chance in the consistent input condition. Neither group (monolingual, bilingual) performed above chance in the variable input condition. Furthermore, there was no main effect of group and interaction between input (consistent/variable) and group, in a generalized linear mixed-effect analysis. Our results for the adults (Study 2) showed that both adult groups performed above chance on the consistent input condition, but only the bilingual group performed above chance in the variable input experiment. Although these findings seem to align with Poepsel and Weiss (2016) proposal that a bilingual advantage is especially prominent in situations where the input contains variability, the interaction between group and experiment version did not reach significance in a mixed-effect regression analysis (*p* = 0.063) without including phonological memory and cognitive control outcomes. Together, these findings speak to the previously reported mixed findings on non-adjacent dependency learning in bilingual compared to monolingual children (Yim and Rudoy, 2013; Verhagen and de Bree, 2021) and statistical learning from variable input (de Bree et al., 2017).

The absence of a robust bilingual advantage in the current study needs to be interpreted in light of some methodological issues. The finding that *t*-tests showed effects for one of the experiment versions only, but the regression analysis showed no clear interaction between group and consistent/variable input suggests that a limitation of our study is that there may have been insufficient power to find an effect. We had not conducted a power analysis prior to conducting this study. Instead, we based our sample size on previous studies into bilingualism and statistical learning. Bartolotti et al. (2011), for instance, collected data from 24 bilinguals, and Wang and Saffran (2014) report data of 24 bilinguals and 24 monolinguals. Our sample sizes were higher than in some earlier studies that used the same designs and reported effects: Poepsel and Weiss (2016) included 17 monolinguals, 17 Chinese-English, and 17 English-Spanish bilinguals and de Bree et al. (2017) included 24 monolinguals and only 14 bilinguals. Yim and Rudoy (2013)'s study consisted of a larger sample (63 monolinguals and 49 bilinguals). However, their sample size might be this large due to the considerable age range of their participants (5 to 13 years). Indeed, age was an important and significant predictor of auditory statistical learning in both groups in their study. Onnis et al. (2018) appears to be the only study on bilingualism and statistical learning in which a power analysis was conducted beforehand to establish that a sample of 55 bilingual (undergraduate) participants was necessary. There are challenges in conducting a priori power analyses, in terms of generalizability across designs and assumptions on which to base the analyses. However, reliance on power analyses is needed in future studies on effects of bilingualism on statistical learning to be more confident about the interpretations.

Our results included a three-way interaction in both studies between group, experiment version (consistent/variable input) and experiment order (consistent input experiment first or second). This interaction indicated that participants were influenced by prior experience with the stimuli in the experiment, even if they completed that experiment one to two weeks earlier, and that this influence differed across monolingual and bilingual groups. Moreover, the direction of the interaction order was different for children and adults, yielding a complex pattern of results. One tentative conclusion is that the bilinguals seemed to be less affected by experiment order than the monolinguals, at least in Study 2. The other interactions with experiment order were both hard to interpret and to relate to previous studies on non-adjacent dependency learning in monolingual and bilingual children: Earlier work using similar tasks with the exact same stimuli (but a different task design) only assessed learning from consistent (and not variable) input (Verhagen and de Bree, 2021), or kept experiment order constant (de Bree et al., 2017), such that all children completed the consistent before the variable input experiment.

The composition of our bilingual groups might also have influenced our results: participants in both studies constituted a group of participants speaking Dutch and a myriad of possible other languages. Furthermore, variability was likely present in language usage and proficiency across participants. Onnis et al. (2018) found that bilingual adults with more balanced proficiency in their two languages learned statistical patterns in two miniature grammars better than bilinguals who were dominant in one of their languages. Since we did not take into account individual differences in the bilingual participants' language dominance and proficiency, a limitation of our study is that we cannot draw any conclusions about the potential effects of these factors. Other sources of variation in our sample (as well as in many of the earlier studies) were the languages spoken by the bilinguals and how typologically similar a bilingual's two languages are. It is not unlikely that the similarity between participants' languages and the language that the artificial language is based on determine the ease with which the artificial language is learned. Also, and more speculatively, it is possible that the typological similarity between a bilingual's two languages mediates learning, such that bilinguals who speak two typologically very different languages develop better metalinguistic abilities (or improved “structural sensitivity,” cf. Kuo and Anderson, 2012; Kuo et al., 2016) that help them extract linguistic structure in statistical learning tasks. However, previous non-adjacent dependency learning studies with mono- and bilingual children that did find effects of bilingualism also contained heterogeneous samples of bilinguals that varied not only in the languages spoken, but also in language use, language proficiency, and age of onset (de Bree et al., 2017; Verhagen and de Bree, 2021), making it unclear to which extent this heterogeneity affected our findings. Future work should take these factors into account.

There is a real possibility that there is no robust across-the-board bilingual advantage in statistical learning, similar to other areas of research on statistical learning (Schmalz et al., 2017; West et al., 2021). Future work might investigate how presentation order of experiments influences the results. Another avenue is to explore whether a bilingual advantage surfaces solely or more prominently when multiple statistical patterns have to be tracked rather than one pattern. Bilingual learners encounter different languages and may therefore encounter more different patterns than monolinguals (depending on their language experiences). While there has been research on bilinguals' tracking of multiple statistical regularities (for an overview, see Weiss et al., 2020), to the best of our knowledge, studies have not yet compared single and multiple pattern tracking within the same participants, while taking prior language experience into account (see also Weiss et al., 2020).

### Statistical learning and phonological memory and cognitive control

Bilinguals did not outperform monolinguals on tasks of phonological memory and cognitive control. In fact, for the adults, we found that the bilinguals performed *less* well on the cognitive control task than the monolinguals. Furthermore, there was no clear association between phonological memory and cognitive control abilities and statistical learning from variable input, once these factors were entered in the regressions. For the adults, cognitive control was positively related to NADL irrespective of whether the input was variable. For the children, no effects of phonological memory or cognitive control emerged.

Overall, our findings are in line with those in earlier work, showing no strong evidence for effects of bilingualism on phonological memory and cognitive control (Paap et al., 2015; van den Noort et al., 2019). Possible explanations relate to the tasks and participants at stake: some studies found that the advantage is mainly seen in complex tasks, and does not show in young adults, the current age group, who are at their peak of EF development (Bialystok et al., 2004). Furthermore, the type of bilingual speakers might have played a role. Earlier work has suggested that the advantage is related to bilinguals who use their languages in specific dual-language contexts, for example, with interlocutors who do not speak both languages, such that switching between languages is required to maintain mutual understanding (Green and Abutalebi, 2013).

Thus, while there are some indications from the current study as well as earlier work (Bartolotti et al., 2011) that bilingualism positively affects statistical learning and that this might be due to enhanced phonological memory and cognitive control, the current results as well as earlier mixed findings (Bartolotti et al., 2011; Verhagen and de Bree, 2021) make it unlikely that enhanced phonological memory and cognitive control impact strongly on statistical learning. Both in our study and in earlier studies, correlations between cognitive abilities and statistical learning were found for only some of the tasks or experiments. This may suggest that correlations are not robust and modulated by specifics of the tasks used, such as the stimuli used, task modality, and presumably also the order in which tasks are administered.

## Conclusion

The present results suggest that there might be some advantage of bilinguals in statistical learning, but this advantage is not robust. It largely surfaced only in *t*-tests against chance for the groups separately, did not surface in the same way for children (where it was found for consistent input) and adults (where it was found for variable input), and was modulated by experiment order. As such, the current results add to the mixed findings in earlier work that indicate that there is no broad, overall effect of bilingualism in statistical learning. They raise the suggestion that future assessment of statistical learning should also take variation within bilingual samples into account. Furthermore, our results provide no evidence that any enhancement in bilinguals' statistical learning was related to improved phonological memory and cognitive control: bilinguals did not outperform monolinguals on these cognitive measures and performance on these measures did not consistently relate to statistical learning outcomes. Taken together, these findings suggest that any potential effects of bilingualism on statistical learning probably do not involve enhanced cognitive abilities associated with bilingualism (Kovács and Mehler, 2009; Kuo and Kim, 2014; Hirosh and Degani, 2017). Future work might explore further to what extent effects, if found, are due to individual differences in bilingual language use, proficiency, and exposure.

## Data availability statement

The datasets presented in this study can be found in online repositories. The names of the repository/repositories and accession number(s) can be found below: https://osf.io/b4ps6/?view_only=a18f5b5cb1d04905b6c26f29de2f43b1.

## Ethics statement

The research was conducted in accordance with The Netherlands Code of Conduct for Scientific Practice, as well as with the guidelines of the Ethics Committees of the University of Amsterdam and Utrecht University. Written informed consent to participate in this study was provided by the participants' legal guardian/next of kin.

## Author contributions

JV: conceptualized the study, set up experiments, analyzed the data, wrote study, and revised the draft. EB: conceptualized the study, set up experiments, wrote parts of study, and revised draft. All authors contributed to the article and approved the submitted version.
