# Supplementary material for: Non-adjacent dependency learning from variable input: investigating the effects of bilingualism, phonological memory, and cognitive control
Source: Front Psychol. 2023 Jul 12;14:1127718. doi: 10.3389/fpsyg.2023.1127718 (PMC10370494; doi:10.3389/fpsyg.2023.1127718)
Supplement: Supplementary file 1 [file Data_Sheet_1.pdf]

Non-adjacent Dependency Learning from Variable Input: Investigating the Effects of Bilingualism, Phonological Memory, and Cognitive Control

Appendices

Appendix A

Table A1

Stimuli of the Forced-Choice Selection Tasks in the Consistent and Variable Input Experiments

	Nr. of items	Stimuli in Language1		Stimuli in Language2	
Consistent input experiment					
trained-untrained	2	a-$X_{(1,2,3,4)}$-b -	a-$X_{(1,2,3,4)}$-d*	a-$X_{(1,2,3,4)}$-b	a-$X_{(1,2,3,4)}$-d*
		(rak X toef)	(rak X lut)*	(sot X toef)	(sot X lut)*
	2	a-$X_{(1,2,3,4)}$-b -	a-$X_{(1,2,3,4)}$-d*	a-$X_{(1,2,3,4)}$-b -	a-$X_{(1,2,3,4)}$-d*
		(rak X toef)	(sot X toef)*	(sot X toef)	(rak X toef)*
	2	c-$X_{(1,2,3,4)}$-d -	c-$X_{(1,2,3,4)}$-d*	c-$X_{(1,2,3,4)}$-d -	c-$X_{(1,2,3,4)}$-d*
		(sot X lut)	(sot X toef)*	(rak X lut)	(rak X toef)*
	2	c-$X_{(1,2,3,4)}$-d -	c-$X_{(1,2,3,4)}$-d*	a-$X_{(1,2,3,4)}$-b -	a-$X_{(1,2,3,4)}$-d*
		(sot X lut)	(rak X lut)*	(rak X lut)	(sot X lut)*
Variable input experiment					
trained-untrained	2	a-$X_{(1,2,3,4)}$-b -	a-$X_{(1,2,3,4)}$-d*	a-$X_{(1,2,3,4)}$-b -	a-$X_{(1,2,3,4)}$-d*
		(rak X toef)	(sot X toef)*	(sot X toef)	(rak X toef)*
	2	c-$X_{(1,2,3,4)}$-d -	c-$X_{(1,2,3,4)}$-d*	a-$X_{(1,2,3,4)}$-b -	a-$X_{(1,2,3,4)}$-d*
		(sot X lut)	(sot X toef)*	(rak X lut)	(rak X toef)*
trained-noise	2	a-$X_{(1,2,3,4)}$-b -	a-$X_{(1,2,3,4)}$-d	a-$X_{(1,2,3,4)}$-b -	a-$X_{(1,2,3,4)}$-d
		(rak X toef)	(rak X lut)	(sot X toef)	(sot X lut)
	2	a-$X_{(1,2,3,4)}$-b -	a-$X_{(1,2,3,4)}$-d	a-$X_{(1,2,3,4)}$-b -	a-$X_{(1,2,3,4)}$-d
		(sot X lut)	(rak X lut)	(rak X lut)	(sot X lut)

Note. *Refers to untrained triplets; X refers to the different X-items used. Note that different, pseudo-randomized ordering of the items was used in the experiments.

Appendix B

Full Results of the Analyses Presented in Study 1

Table B1

Results of the Mixed-Effects Model on Children's Scores on the Consistent and Variable Input NADL Experiments

	Estimate	SE	*z*	*p*
Intercept	0.089	0.102	0.872	.383
Group	-0.250	0.179	-1.400	.162
Experiment version	-0.412	0.184	-2.245	.025
Experiment order	-0.066	0.179	-0.367	.714
Age	-0.002	0.077	-0.027	.979
Group * Experiment version	0.281	0.356	0.788	.431
Group * Experiment order	0.178	0.363	0.492	.623
Experiment version * Experiment order	-0.214	0.357	-0.600	.549
Group * Experiment version * Experiment order	1.624	0.717	2.267	.023

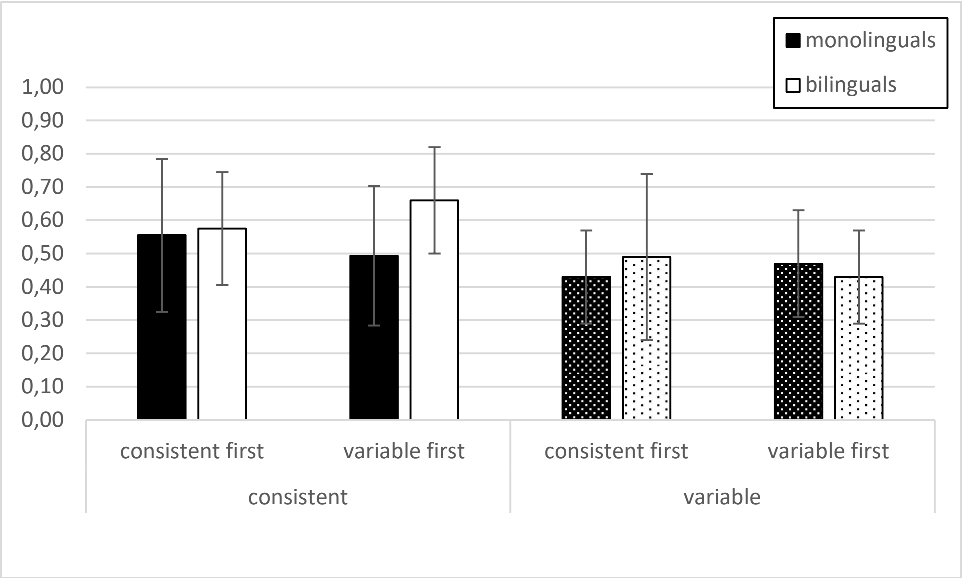

Figure B1.

Proportion Correct per Group in the Consistent and Variable Input NADL Experiments by Experiment Order

Table B2

Results of the Mixed-Effects Model on Children's Scores on the Variable Input NADL Experiment

	Estimate	SE	*z*	*p*
Intercept	-0.208	0.137	-1.512	.130
Group	-0.021	0.205	-0.104	.917
Item type	-0.062	0.229	-0.270	.787
Age	0.060	0.089	0.667	.505
Group * Item type	0.076	0.410	0.186	.853

Table B3

Partial Correlations between Performance on the NADL Experiments and the Nonword Repetition and Flanker Tests for the Monolingual and Bilingual Children (with Age Controlled)

	1.	2.	3.	4.	5.	6.	7.
1. NADL Consistent input (trained-untrained)	-	-.36	-.04	-.10	-.05	.16	.27
2. NADL Variable input (trained-untrained)	-.07	-	.04	-.12	-.33	-49*	-.49*
3. NADL Variable input (trained-noise)	.16	.35	-	.08	-.12	-.02	.18
4. NWR	-.26	.20	-.14	-	.29	.22	-.07
5. Flanker Congruent	.12	.20	.27	-.01	-	.91***	.11
6. Flanker Incongruent	.17	.13	.28	-.09	.95***	-	.51*
7. Flanker Difference score	.19	-.17	.10	-.24	.19	.48*	-

Note. Correlations for the monolingual children are presented above the diagonal; correlations for the bilingual children below the diagonal. * $p < .05$, ** $p < .01$, *** $p < .001$

Table B4

Results of the Mixed-Effects Model on Children's Scores on the Consistent and Variable Input NADL Experiments with Phonological Memory and Cognitive Control as Fixed-Effect Factors

	Estimate	SE	z	p
Intercept	0.091	0.100	0.912	.362
Group	-0.240	0.180	-1.333	.183
Experiment version	-0.410	0.185	-2.220	.027
Experiment order	-0.051	0.181	-0.284	.776
NWR (sum scores)	0.006	0.009	0.655	.512
Flanker (difference scores)	0.071	0.075	0.956	.339
Age	0.003	0.078	0.040	.968
Group * Experiment version	0.254	0.359	0.708	.479
Group * Experiment order	0.218	0.368	0.594	.553
Experiment version * Experiment order	-0.201	0.359	-0.560	.575
Group * Experiment version * Experiment order	1.635	0.722	2.265	.024

Appendix C

Full Results of the Analyses Presented in Study 2

Table C1

Results of the Mixed-Effects Model on Adults' Scores on the Consistent and Variable Input NADL Experiments

	Estimate	SE	*z*	*p*
Intercept	0.526	0.190	2.772	.006
Group	-0.020	0.310	-0.065	.948
Experiment version	-0.311	0.212	-1.466	.143
Experiment order	-0.272	0.309	-0.879	.379
Group * Experiment version	-0.745	0.401	-1.858	.063
Group * Experiment order	0.919	0.616	1.491	.136
Experiment version * Experiment order	-0.391	0.401	-0.977	.329
Group * Experiment version * Experiment order	-1.880	0.803	-2.341	.019

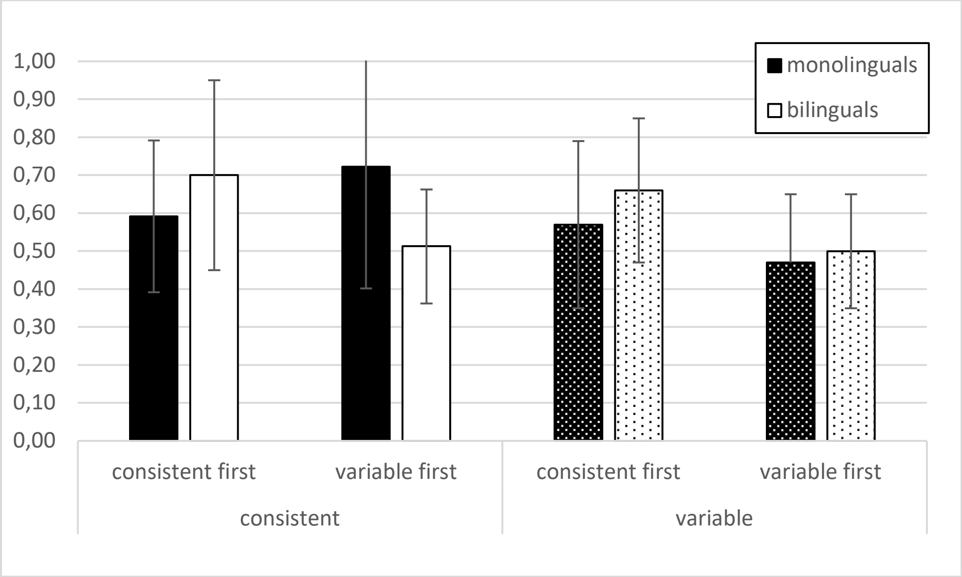

Figure C1.

Proportion Correct per Group in the Consistent and Variable Input NADL Experiments by Experiment Order

Table C2

Results of the Mixed-Effects Model on Children's Scores on the Variable Input NADL Experiment

	Estimate	SE	*z*	*p*
Intercept	0.230	0.125	1.845	.065
Group	-0.226	0.231	-0.977	.328
Item type	0.346	0.215	1.613	.107
Group * Item type	-0.233	0.407	-0.571	.568

Table C3

Bivariate Correlations between NADL, NRT, and TMT Scores

	1.	2.	3.	4.	5.	6.	7.
1. NADL Consistent input (trained-untrained)	-	.32	-.28	-.02	-.19	-.25	-.22
2. NADL Variable input (trained-untrained)	.37	-	-.13	-.40	-.11	-.13	-.11
3. NADL Variable input (trained-noise)	-.27	-.20	-	.19	-.30	-.23	-.15
4. NRT	.21	.13	-.10	-	-.06	-.27	-.31
5. TMT Part A	-.27	.03	-.02	-.21	-	.68**	.40*
6. TMT Part B	-.44*	-.06	.06	-.56**	.74***	-	.94***
7. TMT Difference Score	-.42*	-.10	.10	-.61**	.39*	.91***	-

Note. Correlations for the monolinguals are presented above the diagonal, correlations for the bilinguals below the diagonal. * $p < .05$, ** $p < .01$, *** $p < .001$

Table C4

Results of the Mixed-Effects Model on Adults' Scores on the Consistent and Variable Input NADL Experiments with Phonological Memory and Cognitive Control as Fixed-Effect Factors

	Estimate	SE	*z*	*p*
Intercept	0.441	0.185	2.380	.017
Group	-0.301	0.303	-0.995	.320
Experiment version	-0.363	0.219	-1.657	.098
Experiment order	-0.673	0.307	-2.193	.028
TMT (difference score)	-0.237	0.106	-2.232	.026
NWR (sum score)	-0.016	0.015	-1.070	.285
Group * Experiment version	-0.897	0.416	-2.156	.031
Group * Experiment order	0.749	0.597	1.255	.210
Experiment version * Experiment order	-0.698	0.413	-1.688	.091
Group * Experiment version * Experiment order	-1.733	0.831	-2.085	.037
